# Hyperbaric Oxygen Therapy Combined With Standard Wound Care Versus Standard Wound Care Alone in Patients With Diabetic Foot Ulcers: A Prospective Comparative Study

**DOI:** 10.7759/cureus.74964

**Published:** 2024-12-02

**Authors:** Aldrin L Myrthong, Shrutika Gurav, Suresh Mahankudo, Kashif F Ansari, Manasi Sawant, Kaushal Lahoti

**Affiliations:** 1 General Surgery, Grant Government Medical College and Sir JJ Group of Hospitals, Mumbai, Mumbai, IND

**Keywords:** amputation, diabetes, diabetic foot ulcer, hyperbaric oxygenation, wound healing

## Abstract

Background

Non-healing diabetic foot ulcers (DFUs) are significant risk factors for amputations. Though the available literature suggests that adjuvant hyperbaric oxygen therapy (HBOT) fastens the healing process and reduces the risk of amputations, its overall evidence in the reduction of amputation remains controversial. Thus, the present study aimed to compare the efficacy and safety of adjuvant HBOT and standard wound care (SWC) with SWC alone in patients with DFUs.

Methods

This prospective, randomized, controlled study involved 60 adult patients with DFU. Based on the simple random number table, the patients were equally randomized into two group: adjuvant HBOT and SWC (n=30) with SWC alone (n=30). The patients received 24 sessions (six sessions per week) of HBOT (3.0 absolute atmospheric pressure) for 45 minutes daily over a period of four consecutive weeks. The outcome measures included wound size reduction, wound bed condition, complications, and proportion of patients undergoing amputation. The patients were assessed at four-week follow-up.

Results

At four weeks, both the groups had a significant reduction in pain score, wound size, and inflammation of the surrounding skin compared to baseline (all p<0.001). At the end of the study, the adjuvant HBOT and SWC group had significantly reduced pain score and wound size as well as a greater proportion of healthy granulation tissue in the wound bed relative to the SWC group (all p=0.001). Moreover, adjuvant HBOT and SWC led to a significantly reduced incidence of minor amputation (p=0.001), while complications were comparable between the groups (p=0.198).

Conclusion

Adjuvant HBOT and SWC are more effective than SWC in healing the DFUs and reduction of minor amputations.

## Introduction

Diabetes results in a great economic burden, mainly owing to various complications that lead to longer hospital stays, amputation, and decreased quality of life [1]. Globally, around 33% of patients with diabetes develop diabetic foot ulcers (DFUs), affecting approximately 18.6 million individuals per year. About 50% of these ulcers get infected, and around 20% of these infected ulcers lead to amputation of a part or the whole foot [2].

In patients with DFU, hyperbaric oxygen therapy (HBOT) is reported to enhance local tissue oxygen supply, reduce tissue hypoxia, and decrease wound infection by exhibiting antibacterial properties [3]. By increasing tissue oxygen levels, HBOT accelerates the healing process of ulcers and prevents amputations [4].

A meta-analysis reported that HBOT significantly increases the healing rate of DFUs, reduces the healing time, and decreases the chances of major amputation (above-ankle joint amputation) [5]. Another recent meta-analysis demonstrated that HBOT is significantly superior to other treatment modalities for wound healing rates as well as minor (below ankle or toe/forefoot amputation) and major amputations [6]. Similarly, other meta-analyses reported that HBOT was significantly effective in complete healing of DFUs and decreased the incidence of major amputation, though it was not effective for minor amputations and led to higher adverse events (oxygen toxicity (O2 induced seizure), ocular effects, barotraumatic lesions, injury to ear, hypoglycemia, and cataract) relative to standard treatment [4,7].

The variation in the outcomes associated with HBOT compelled us to perform this study. Moreover, the duration, frequency and number of cycles of HBOT are not defined. However, in our resource constraint setting, we use HBOT of reduced duration and cycle, which produces a similar effect as mentioned in the literature [8]. Thus, the aim of this study was to validate the effectiveness of HBOT with standard wound care (SWC) compared with SWC alone in terms of wound size reduction, wound bed condition, complications, and proportion of patients undergoing amputation in patients with DFUs.

## Materials and methods

This prospective comparative study was performed in the Department of General Surgery of a tertiary care institute over a period of 18 months (September 2022 to February 2024). The study included adult diabetic in-patients aged 18 years or more, either sex, presenting with non-healing DFUs for at least one month despite standard of care as well as those with gangrenous foot and normal major vessels Doppler findings.

While, the patients with DFUs or gangrenous foot attributed to causes other than diabetes; those with osteomyelitis and peripheral vascular disease with major vessel occlusion; those with contraindication for HBOT, including active cancerous condition, untreated pneumothorax, pulmonary emphysema with retention of CO2, and chronic obstructive pulmonary disease; patients receiving amphetamine, thyroid hormone, corticosteroids, or catecholamine; and those planned for vascular surgical procedures or revascularization of the limb were excluded. Moreover, pregnant and lactating women were also excluded. Prior to study initiation, the study was approved by the Institutional Ethics Committee, and written informed consent was obtained from all the patients.

**Figure 1:**
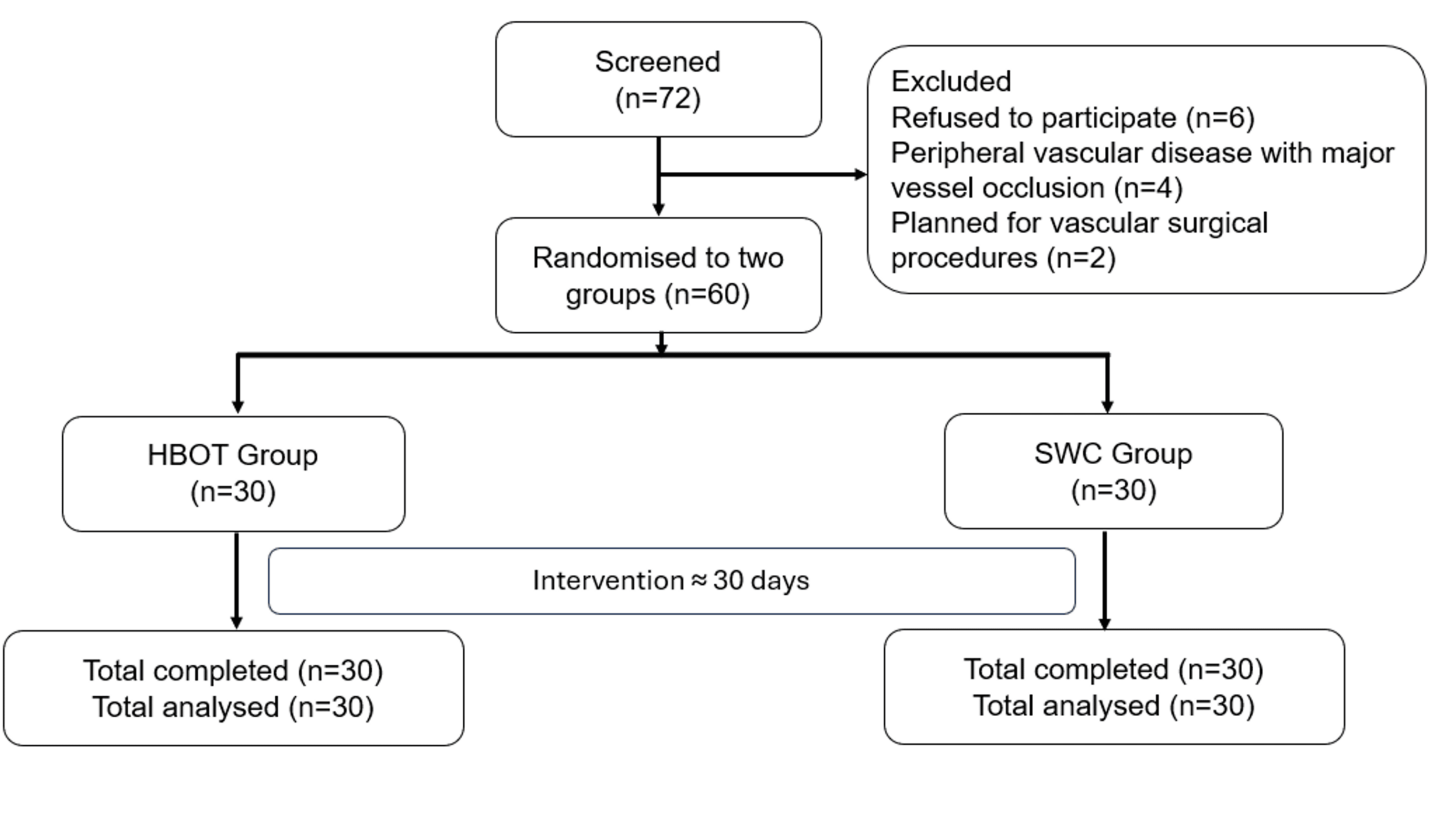
Study flow diagram HBOT: Hyperbaric oxygen therapy; SWC: Standard wound care

Following the eligibility assessment, a total of 60 patients were enrolled in the study. Based on the simple random number table, the patients were divided into two groups: HBOT (100%) + SWC (HBOT, n=30) and SWC (SWC, n=30). Figure 1 illustrates the study flow diagram.

SWC involved topical and systemic therapy, and included maintenance of adequate blood glucose control, debridement of necrotic tissue, off-loading, antibiotic therapy for infected DFUs, and topical dressings, according to the grade of the ulcer. HbA1c was performed to assess diabetes control and total leucocyte count (TLC) was used as a marker of infection. The wound swabs were sent for culture and antibiotic sensitivity at the start of the study. DFU microbiology and the presence of infection were assessed through wound cultures collected with the aseptic swab technique and subjected to aerobic and anaerobic bacterial cultures. The use of antibiotics was based on the departmental protocol as well as culture and sensitivity findings.

All the hospitalized patients with DFU received HBOT in a single-person chamber. Primary mechanical debridement was done when required. HBOT was given daily after the debridement as well. The patients were placed in a compression chamber and treated with 3.0 absolute atmospheric pressure for 45 minutes daily. Each patient received 24 sessions over a period of four consecutive weeks, six days a week. The mean period of the intervention was around 30 days. However, as soon as the patients improved symptomatically, they were discharged and the remaining HBOT sessions were given on an OPD basis.

Outcome measures

Various outcome measures were used to assess the efficacy of HBOT as adjunctive therapy for patients with non-healing DFU. The outcome measures included wound size reduction, wound bed condition, complications, and the proportion of patients undergoing amputation. The outcomes were evaluated during the treatment period and at a four-week follow‑up. Both the study groups were compared in terms of all the outcome measures.

Overall, DFUs were regarded to be healed when they were completely covered by epithelial tissue. While, Wagner grade IV ulcers were regarded to be healed, when the gangrene had completely separated, and underlying ulcers were completely covered by regenerating epithelial tissue. However, the ulcer was regarded to be not healed, if the patient underwent major (above ankle) amputation. The vascular surgeon was responsible for the decision regarding amputation, if the wound fulfilled any of the criteria: no bear weight on the involved limb and pain leading to severe disability, current risk of severe systemic infection associated with the wound, and deep infection of bone and tendons. DFU-related pain was evaluated on the visual analogue scale (score range 0 to 10), with 0 and 10 suggesting no and worst imaginable pain, respectively.

Sample size calculation

The estimated sample size was based on the efficacy in terms of the rate of wound healing between the two groups. With reference to a previous study [8], it was observed that 73.3% of patients healed with HBOT, and 13.3% of patients healed with SWC. Thus, the sample size of 30 patients per group provided a 90% power for detecting a significant difference between the two groups at an alpha level of 0.05 with a relevant clinical difference of a minimum of 40% in the rate of wound healing between the two groups. The sample size is derived from the following formula:

\[ \frac{\left[ Z_{(1-\alpha/2)} \cdot \sqrt{2P(1-P)} + Z_{(1-\beta)} \cdot \sqrt{P_1(1-P_1) + P_2(1-P_2)} \right]^2}{(P_1 - P_2)^2}\]

Where, Zα/2 is the critical value of the normal distribution at α/2 (for a confidence level of 95%, α is 0.05 (one-sided and the critical value is 1.645)), Zβ is the critical value of the normal distribution at β (for a power of 80%, β is 0.2 and the critical value is 0.842), and P1 and P2 are the expected sample proportions of the two groups.

Statistical analyses

The data was analyzed using Statistical Package for the Social Sciences (IBM SPSS Statistics for Windows, IBM Corp., Version 28.0, Armonk, NY). The categorical and continuous variables are represented as frequency (percentage) and mean (standard deviation, SD), respectively. The chi-square test and independent sample t-test were used to assess the association between categorical and continuous variables, respectively. Moreover, the chi-square test and paired t-test were used to assess within-group differences in continuous variables, respectively. A two-tailed probability value of < 0.05 was considered as statistically significant.

## Results

In both groups, there were no dropouts during the study period, and all the enrolled patients completed the study. At baseline, both groups were comparable regarding age (p=0.951), sex (p=0.417), BMI (p=0.939), duration of diabetes mellitus (DM) (p=0.111), interventions (including insulin, and/or oral hypoglycemic agents (OHA), p=0.985), injury mechanism (p=0.811), hypertension (p=1.000), history of alcohol intake (p=0.774), and history of tobacco intake (p=0.876) (Tables 1-2). In HBOT and SWC groups, most of the patients had controlled diabetes (83.33% and 80%, respectively), and were on OHA and/or insulin. While only a few patients had uncontrolled diabetes (16.67% and 20%, respectively), requiring initiation of insulin therapy, glycemic control was achieved in three to four days.

**Table 1:** Comparison of demographic characteristics SD: Standard deviation; HBOT: Hyperbaric oxygen therapy; SWC: Standard wound care; Data represented as n (%) and mean (SD); p <0.05 is considered significant.

Characteristics	HBOT (n=30)	SWC (n=30)	Statistical test	p-value
Age, years, mean (SD)	57.77 (13.71)	56.57 (11.83)	Independent sample t-test	0.951
Sex, n (%)				
Male	18 (60)	21 (70)	Chi-square test	0.417
Female	12 (40)	9 (30)		
BMI, kg/m^2^, mean (SD)	29.09 (3.92)	29.17 (3.5)	Independent sample t-test	0.939
Hypertension, n (%)	22 (73.3)	22 (73.3)	Chi-square test	1.000
History of alcohol intake, n (%)	9 (30.0)	8 (26.7%)	Chi-square test	0.774
History of tobacco intake, n (%)				
Bidi smoker	6 (20.0)	7 (23.3)	Chi-square test	0.876
Cigarette smoking	2 (6.7)	2 (6.7)		
Denies tobacco use	12 (40.0)	9 (30.0)		
Oral tobacco chewer	10 (33.3)	12 (40.0)		

**Table 2:** Comparison of clinical characteristics DM: Diabetes mellitus; HBOT: Hyperbaric oxygen therapy; OHA: Oral hypoglycemic agents; SD: Standard deviation; SWC: Standard wound care; Data represented as n (%) and mean (SD); p <0.05 is considered significant.

Characteristics	HBOT (n=30)	SWC (n=30)	Statistical test	p-value
Interventions, n (%)				
Insulin mixtard	2 (6.7)	2 (6.7)	Chi-square test	0.985
Regular insulin	14 (46.7)	14 (46.7)		
Regular Insulin + OHA	9 (30.0)	8 (26.7)		
OHA	5 (16.7)	6 (20.0)		
Duration of DM, yrs, mean (SD)	16.80 (5.48)	14.57 (5.65)	Independent sample t-test	0.111
Injury mechanism, n (%)				
Animal bite	4 (13.3)	3 (10.0)	Chi-square test	0.811
Bed sore	6 (20.0)	3 (10.0)		
Self-scratch	1 (3.3)	1 (3.3)		
Spontaneous	3 (10.0)	3 (10.0)		
Trivial trauma	16 (53.3)	20 (66.7)		

Comparison of laboratory characteristics revealed no significant difference between the groups regarding hemoglobin (p=0.782), TLC (p=0.206), serum proteins levels (p=0.509), HbA1c (p=0.873), and wound culture (p=0.547). However, the length of hospital stay was significantly greater in the HBOT group relative to the SWC group (p=0.001) (Table 3).

**Table 3:** Comparison of laboratory characteristics and hospital stay Hb: Hemoglobin; HbA1c: Glycated hemoglobin; HBOT: Hyperbaric oxygen therapy; SD: Standard deviation; TLC: Total leucocyte count; SWC: Standard wound care; Data represented as n (%) and mean (SD); p <0.05 is considered significant.

Characteristics	HBOT (n=30)	SWC (n=30)	Statistical test	p-value
Hb, g%, mean (SD)	11.11 (1.46)	11.21 (1.55)	Independent sample t-test	0.782
TLC, × 10^9^/L, mean (SD)	12.4 (4.45)	11.06 (3.62)	Independent sample t-test	0.206
Serum proteins, g/dL, mean (SD)	5.12 (1.76)	4.82 (1.75)	Independent sample t-test	0.509
HbA1c, %, mean (SD)	8.42 (1.42)	8.48 (1.45)	Independent sample t-test	0.873
Wound swab, n (%)				
No organism found	3 (10.0)	7 (23.3)	Chi-square test	0.547
Acinetobacter	12 (40.0)	11 (36.7)		
Escherichia coli	2 (6.7)	3 (10.0)		
Klebsiella pneumoniae	5 (16.7)	2 (6.7)		
Proteus mirabilis	1 (3.3)	2 (6.7)		
Pseudomonas aeruginosa	3 (10.0)	4 (13.3)		
Staphylococcus aureus	2 (6.7)	1 (3.3)		
Streptococcus viridans	2 (6.7)	0 (0)		
Hospital stay, days, mean (SD)	14.97 (4.46)	8.77 (3.84)	Independent sample t-test	0.001

Comparison of pain and ulcer characteristics revealed that groups were comparable regarding baseline pain score (p=0.796), size of ulcer (p=0.359), wound bed (p=0.223), discharge (p=0.551), and surrounding skin (p=0.134). Within-group analysis suggested that pain score, ulcer size, and inflammation of the surrounding skin decreased significantly in both the groups at four weeks compared to baseline (all p<0.001). At four weeks, the between-group analysis revealed that pain score and ulcer size decreased significantly in the HBOT group relative to the SWC group (both p=0.001). Moreover, a significantly greater proportion of patients in the HBOT group had healthy granulation tissue in the wound bed relative to the SWC group (p=0.001). However, both groups did not differ in wound discharge characteristics (p=0.058) and inflammation of the surrounding skin (p=0.832) (Tables 4-5).

**Table 4:** Comparison of pain and ulcer characteristics HBOT: Hyperbaric oxygen therapy; SD: Standard deviation; SWC: Standard wound care; Data represented as n (%) and mean (SD); p <0.05 is considered significant.

Outcomes		HBOT (n=30)	SWC (n=30)	Statistical test	p-value
Pain score, mean (SD)					
Baseline		8.27 (1.70)	8.30 (1.39)	Independent sample t-test	0.796
4-weeks		2.53 (2.16)	6.03 (1.75)		0.001
p-value		<0.001	<0.001		
Size of ulcer, mean (SD)					
Baseline		6.15 (2.53)	6.68 (1.83)	Independent sample t-test	0.359
4-weeks		3.00 (2.38)	5.22 (2.11)		0.001
p-value		<0.001	<0.001		
Wound bed, n (%)					
Baseline	Exposed tendons	8 (26.7)	11 (36.7)	Chi-square test	0.223
	Necrotic soft tissue	11 (36.7)	13 (43.3)		
	Subcutaneous tissue	3 (10.0)	4 (13.3)		
	Unhealthy granulation tissue	8 (26.7)	2 (6.7)		
4-weeks	Exposed bone	0 (0.0)	2 (6.7)	Chi-square test	0.001
	Exposed tendons	1 (3.3)	16 (53.3)		
	Healthy granulation tissue	29 (96.7)	12 (40.0)		

**Table 5:** Comparison of ulcer characteristics HBOT: Hyperbaric oxygen therapy; SWC: Standard wound care; Data represented as n (%); p <0.05 is considered significant.

Outcomes		HBOT (n=30)	SWC (n=30)	Statistical test	p-value
Discharge, n (%)					
Baseline	Necrotic debris	5 (16.7)	4 (13.3)	Chi-square test	0.551
	Purulent discharge	24 (80.0)	26 (86.7)		
	Slough	1 (3.3)	0 (0.0)		
4-weeks	Greenish discharge	5 (16.7)	4 (13.3)	Chi-square test	0.058
	Necrotic debris	3 (10.0)	6 (20.0)		
	Purulent discharge	0 (0.0)	6 (20.0)		
	Serous discharge	20 (66.7)	12 (40.0)		
	Slough	2 (6.7)	2 (6.7)		
Surrounding skin, n (%)					
Baseline	Mildly inflamed	3 (10.0)	0 (0.0)	Chi-square test	0.134
	Normal	1 (3.3)	3 (10.0)		
	Severely inflamed	26 (86.7)	27 (90.0)		
4-weeks	Mildly inflamed	17 (56.7)	15 (50.0)	Chi-square test	0.832
	Normal	11 (36.7)	12 (40.0)		
	Severely Inflamed	2 (6.7)	3 (10.0)		
Statistical test		Paired t-test	Paired t-test		
p-value		<0.001	<0.001		

Further comparison suggested that a significantly greater proportion of patients in the SWC group had amputation (p=0.001). However, the groups were comparable in terms of complication (p=0.198) (Table 6).

**Table 6:** Comparison of amputation and complications HBOT: Hyperbaric oxygen therapy; SWC: Standard wound care; Data represented as n (%); p <0.05 is considered significant.

Characteristics	HBOT (n=30)	SWC (n=30)	Statistical test	p-value
Amputation, n (%)				
No amputation	26 (86.7)	4 (13.3)	Chi-square test	0.001
Chopart amputation	3 (10.0)	6 (20.0)		
Lisfranc amputation	1 (3.3)	6 (20.0)		
Ray amputation	0 (0.0)	14 (46.7)		
Complications, n (%)				
None	27 (90.0)	25 (83.3)	Chi-square test	0.198
Bleeding	0 (0.0)	2 (6.7)		
Ear barotrauma	1 (3.3)	0 (0.0)		
Lung barotrauma	1 (3.3)	0 (0.0)		
Sepsis	1 (3.3)	3 (10.0)		

## Discussion

In continuation with the evolving literature, the principal findings of the present study suggest that both HBOT combined with SWC and SWC alone led to significant improvement in pain score, wound size, and inflammation of the surrounding skin at the end of the study. However, at the end of the study, HBOT combined with SWC led to a significantly greater decrease in pain score and wound size as well as a significantly greater proportion of patients who developed healthy granulation tissue in the wound bed compared to SWC alone. Moreover, HBOT combined with SWC led to a significantly lesser incidence of amputation, while groups were nearly identical regarding complications.

Around three-fourths of the patients with DFUs report wound-related pain. Pain is reported irrespective of the time of the day, activity, or change in dressing, and adversely affects sleep. Moreover, various authors have observed no significant difference in pain intensity between neuropathic and neuro-ischemic wounds as well as type and triggers of wound-related pain [9]. In these patients, the onset of pain suggests limb-threatening complications, including deep infection and critical ischemia. Moreover, the presence of excess moisture is linked to pain, which can result in macerated callus around the wound edge, thereby promoting the proliferation of bacteria and increasing pain [10]. Both human and animal studies have suggested that HBOT reduces the pain score and pain-related symptoms significantly [10,11]. HBOT reduces pain by various mechanisms. Primarily, it causes a significant rise in partial pressure of tissue O2 (hyperoxia). It also leads to a rise in hydrostatic pressure that triggers arteriolar vasoconstriction, thus relieving tissue edema, without any adverse impact on the effect of hyperoxia. Secondarily, it increases the production of reactive oxygen and nitrogen species as well as inhibits the production of inflammatory mediators [11].

In the present study, we demonstrated that HBOT (at 3.0 absolute atmospheric pressure for 45 minutes daily) combined with SWC led to a significant reduction in ulcer size compared to those receiving SWC alone. The response was clinically evident after 12 sessions and became statistically significant after 24 sessions. The patients in the SWC alone group also had a reduction in ulcer size though the rate of reduction was much slower; this is supported by the finding that a significantly greater proportion of patients receiving adjunctive HBOT developed healthy granulation tissue in the wound. Similarly, other studies have reported that adjunctive HBOT produces a significant reduction in DFU size [12,13]. Moreover, a recent meta-analysis demonstrated that patients receiving adjunct HBOT had a significantly higher chance of DFU healing and these patients had a greater percentage of DFU area reduction following two weeks of therapy [14]. Contrarily, an earlier meta-analysis suggested that HBOT and standard treatment did not differ significantly in the reduction of wound size [4]. Thus, the findings of the present study clearly demonstrated the benefits of HBOT in the reduction of DFU area; however, further research is required to reach a definite conclusion.

In patients with non-healing DFUs, the presence of deep infection, poor glycemic control, neuroischemic foot, and chronic arterial insufficiency are recognized risk factors for amputation. HBOT acts by facilitating physiological effects resulting in reduced regional and local ischemia. Thus, HBOT promotes oxygen-associated mechanisms to improve wound repair and stem cell development in the bone marrow and enhances host antimicrobial responses. In the present study, none of the patients had major amputations, while adjuvant HBOT led to significantly reduced minor amputations than SWC. A recent meta-analysis concluded that HBOT significantly reduces the rate of amputations [7]. Another meta-analysis demonstrated that HBOT leads to a significant reduction in both major and minor amputations [6]. Other meta-analyses found that only major amputations were significantly reduced, while minor amputations were not affected [5,14]. Moreover, another meta-analysis found no significant reduction in major and minor amputations with HBOT compared to standard treatment [4]. Thus, the effect of HBOT on amputations requires further evaluation.

Despite the use of an active comparator arm, the present study had certain limitations. First, the sample size was relatively small. Second, the duration of follow-up was less, and thus, patients could not be assessed till complete healing of DFUs. Third, this was a single-center study, and the findings cannot be generalized.

## Conclusions

To conclude, in patients with non-healing DFUs, adjuvant HBOT and SWC are more effective than SWC in healing the wound and reducing minor amputations. Adjuvant HBOT increases the rate of DFU healing by reducing the wound size and surrounding inflammation as well as promoting regeneration of the granulation tissue. Moreover, complications were comparable with adjuvant HBOT and SWC alone. However, large, randomized trials with longer follow-ups are required to confirm the role of adjuvant HBOT in the reduction of amputations.
